# Development and validation of a reversed-phase HPLC-UV method for simultaneous determination of levosulpiride and omeprazole in human plasma: Applicability of the method for evaluation of pharmacokinetic drug-drug interactions

**DOI:** 10.1371/journal.pone.0309453

**Published:** 2024-08-29

**Authors:** Muhammad Hashim, Lateef Ahmad, Amjad Khan, Muhammad Faheem

**Affiliations:** 1 Department of Pharmacy, University of Swabi, Swabi, Pakistan; 2 Department of Pharmacy, Kohat University of Science and Technology (KUST), Kohat, Pakistan; Icahn School of Medicine at Mount Sinai Department of Pharmacological Sciences, UNITED STATES OF AMERICA

## Abstract

Levosulpiride and omeprazole are co-prescribed for gastrointestinal disorders associated with depression and anxiety. Objective of the study was to develop a sensitive, robust and simple method for simultaneous analysis of levosulpiride and omeprazole in human plasma and applicability of the method in determination of pharmacokinetics drug-drug interaction. In the presented study, a reversed-phase HPLC-UV method was developed for the simultaneous determination of levosulpiride and omeprazole using pantoprazole as the internal standard. Experimental conditions were optimized and the developed method was validated as per standard guidelines (USP and ICH). Furthermore, the developed method was applied for evaluation of pharmacokinetics drug-drug interaction between levosulpiride (50 mg) and omeprazole (40 mg) in healthy human volunteers. Sharpsil C8 column (4.6 × 250 mm, 5 μm), Ultisil C8 column (4.6 mm × 150 mm, 5 μm) and Agilent C18 column (4.6 × 250 mm, 5 μm) were evaluated as stationary phase. The best resolution was achieved with Agilent C18 (4.6 x 250 mm, 5 μm) column and was selected for further study. The mobile phase consisted of a mixture of acetonitrile and phosphate buffer (pH 7.2) in 60:40 by volume, and was pumped at a flow rate of 1 mL/min. Detector wavelength was set at 280 nm. Levosulpiride and omeprazole were extracted from human plasma with ethyl acetate and dichloromethane (4:1, v/v). The calibration curves for both levosulpiride (5–150 ng/mL) and omeprazole (10–1500 ng/mL) were linear. The lower limit of quantification and limit of detection for levosulpiride were 5 and 2 ng/mL, while for omeprazole these were 10 and 3 ng/mL, respectively. Pharmacokinetics analysis showed that co-administration of omeprazole increased the AUC and C_max_ of levosulpiride, while the clearance was reduced. Both the changes were insignificant. Similarly, no significant change in the pharmacokinetic parameters of omeprazole was observed with co-administration of levosulpiride.

## Introduction

The prevalence of gastrointestinal (GIT) diseases is high throughout the world [1] and more than 40% of people are affected throughout the world. The major GIT diseases include gastro-esophageal reflux disorder (GERD), dyspepsia, peptic ulcers, and irritable bowel syndrome. In 2012, in the USA about 8.9 million hospital visits were due to GERD [2]. Functional gastrointestinal disorders also known as gut-brain disorders, which are mainly due to stress and anxiety, have produced a negative impact on the economy and lifestyle [3]. Functional GIT disorders are commonly treated with proton pump inhibitors (PPIs), H_2_-receptor antagonists, prostaglandin analogs, antacids, sucralfate, bismuth compounds, prokinetic agents, etc. [4]. Among PPIs omeprazole (OMP) is the prototype and the mostly prescribed drug [5]. After oral administration, OMP is metabolized by CYP2C19 and CYP3A4 in the liver which makes it vulnerable to drug-drug interactions [6, 7].

PPIs have been successfully used to alleviate the symptoms of GERD and dyspepsia, however, depression and anxiety may reduce the effectiveness of PPIs. Furthermore, reducing the stress has been reported to be helpful in the management of GERD and dyspepsia [8, 9] and in most of the cases GIT disturbances are treated with dual therapy. Levosulpiride (LSP) in combination with PPIs, has been effectively used in the treatment of irritable bowel syndrome, duodenal, and gastric ulcers, caused by depression, stress, schizophrenia, and psychopathology of senescence [10, 11]. After oral administration, it is absorbed from the intestine and excreted unchanged through the kidneys [12]. Among PPIs, OMP is commonly co-prescribed with LSP. LSP is a substrate for p-glycoprotein while OMP is its inhibitor [13], and there is a possibility that these two drugs may alter pharmacokinetics of each other, with subsequent variability in therapeutic response.

A number of HPLC methods have been reported for the analysis of OMP and its metabolites [14]. Similarly, liquid chromatography-mass spectrometry (LCMS) has been utilized for pharmacokinetics of LSP [15, 16]. In one of the study LCMS/MS has been used for simultaneous determination of esomeprazole, rabeprazole, and LSP but the authors had used the same calibration levels for all the three analytes [17]. Technically, Cmax and AUC of LSP are less than OMP and range of the calibration curve should be narrow for LSP. So far, HPLC method has not been reported for simultaneous analysis of OMP and LSP in biological samples. In this study, a reversed phase HPLC-UV method was developed and validated for the simultaneous determination of LSP and OMP in human plasma and pharmaceutical samples. Furthermore, the developed method was applied for the evaluation of pharmacokinetic drug-drug interactions between LSP and OMP and to find out whether dose adjustment is needed when these two drugs are co-administered.

## Materials and methods

### Material

Omeprazole (OMP), levosulpiride (LSP), and pantoprazole sodium (PTP) were obtained from Medizan Laboratories Pvt. Ltd. Islamabad, Pakistan. Potassium dihydrogen phosphate, orthophosphoric acid, HPLC grade methanol, and acetonitrile were purchased from Merck, Germany. Millipore (Milford, USA), distillation apparatus was used for the preparation of distilled water. LSP 50 mg tablets and OMP 40 mg capsules were purchased from a local pharmacy at Peshawar, Pakistan.

### Instrumentation

HPLC system (Model LC-20; Shimadzu, Japan) was consisted of a UV detector (SPD -20AV), autosampler (SIL- 10AF), Column oven (CTO-20A), and a degassing unit (DGU-20ASR). The chromatograms were interpreted by the software Lab Solutions.

### Ethical consideration of the study

The study was conducted according to the “World Medical Association Declaration of Helsinki-Ethical principles of medical research involving human subjects” and its amendments. This study was approved by the ethical committee, Department of Pharmacy, University of Swabi, Swabi, Pakistan (Application number F.NO.UOS/2022/Pharm.02). the study started on 4^th^ June, 2023 and completed on 15^th^ August, 2023.

### Sample preparation

The stock solutions of OMP and LSP were prepared by dissolving in acetonitrile to concentration of 100 μg/mL. PTP was used as the internal standard (IS) and its solution was prepared with a final concentration of 50 μg/mL. The primary stock solution of analytes and internal standard were stored at –20 °C and diluted on need basis. Standard samples were prepared by diluting the stock solution with mobile phase on need basis.

For preparation of plasma samples, blood was collected from human volunteer and centrifuged to separate the cellular components. Plasma was de-proteinated with acetonitrile (two times volume of plasma) and was used for spiking the analytes. A liquid-liquid extraction method, reported by Jin et al., [18], with slight modification was used for extraction of LSP and OMP. Known amount of LSP, OMP and IS were added to plasma (1 mL), and vortexed for 30 sec. 4 ml of extraction solvent (mixture of ethyl acetate and dichloromethane in 4:1 by volume) was added, vortexed for 2 min and centrifuged at 3000 × g for 10 min. The supernatant (organic) layer was separated and dried under a gentle stream of nitrogen gas. The dried residue was reconstituted with 0.2 mL of mobile phase and an aliquot (20 μL) was injected into the HPLC system. Blank and zero samples were also treated in the same manner.

### Optimization of chromatographic conditions

Stationary phase was selected by evaluating different columns including Sharpsil C8 column (4.6 × 250 mm; 5μm), Ultisil C8 column (4.6 mm × 150 mm; 5 μm) and Agilent C18 column (4.6 × 250 mm; 5 μm) were evaluated and the one showing better peak characteristics (peak area, peak height and resolution) was selected for further analysis. Different experimental conditions of HPLC like composition of mobile phase, flow rate of the mobile phase, pH of the mobile phase, detector wavelength, and column oven temperature were optimized as per standard protocols [20].

### Method validation

The developed HPLC method for simultaneous analysis of LSP and OMP was validated for different parameters like linearity, accuracy, precision, sensitivity, repeatability, and stability of samples, as per standard guidelines described in ICH guideline M10 on bioanalytical method validation and study sample analysis [19].

#### Selectivity and specificity

Selectivity of the method was evaluated by analyzing the blank samples (pharmaceutical and plasma sample) and samples containing both the analytes and IS, to observe peak of the endogenous compounds or other interfering component, which affects the retention times and other peak characteristics of the analytes and IS. The specificity of the method was performed to detect and differentiate between the metabolites and the analytes in the samples, collected at different time intervals from healthy volunteers after administration of both drugs. Furthermore, the resolution between the two consecutive peaks was also calculated.

#### Linearity

The linearity of the HPLC method was determined by analyzing the pharmaceutical samples (samples prepared in the mobile phase) and the plasma samples (plasma spiked with mixture of LSP and OMP). Working solutions were prepared at six different concentration levels of LSP (5, 10, 25, 50, 100, and 150 ng/mL) and OMP (10, 100, 200, 500, 1000, and 1500 ng/mL) by diluting with mobile phase and fixed quantity of internal standard (200 ng/mL) was added in each solution. A curve was constructed by plotting peak response ratio (ratio of the peak area of the analyte to IS) of each analyte. Regression analysis of the curves was performed and correlation coefficient (r^2^) and the equation for a straight line (*y* = *a* + *bx*) were determined using MS Excel, 2016. The calibration standards were back-calculated to determine the total error (percent error).

#### Sensitivity

Sensitivity of the method was assessed through the lower limit of quantification (LLOQ) and the lower limit of detection (LLOD), determined on the basis of signal to noise ratio. LLOQ was the concentration at which the response of the analytes was 10 times of the highest noise peak while LLOD was the concentration of analytes that produced a response equivalent to three times that of the noise.

#### Accuracy

The accuracy (percent recovery of the measured concentration) was determined at four different concentration levels including LLOQ, low quality control (LQC), medium quality control (MQC), and high-quality control (HQC). The corresponding concentrations of these four levels were 5, 10, 50, and 120 ng/mL for LSP and 10, 30, 500, and 1200 ng/mL, of OMP. Each sample was analyzed in 5 replicates (n = 5) and their mean, standard deviation (SD) and %RSD were calculated.

#### Precision

Precision studies included repeatability and intermediate precision. Repeatability was determined in terms of injection repeatability and analysis repeatability. Injection repeatability was determined by observing the peak areas and retention times by injecting 10, 50, and 100 ng/ml of LSP and 100, 500, and 1000 ng/ml of omeprazole (n = 5). Analysis repeatability involved the amount recovered from the spiked samples at four concentration levels i.e. LLQC, LQC, MQC, and HQC. Intermediate precision was determined from the intra-day and inter-day repeatability. The spiked plasma samples containing both levosulpiride and omeprazole at four different concentration levels were injected into the HPLC system at different times of the day (9:00, 14:00, and 22:00 hours) for 3 days and the amount recovered from the spiked samples was considered for intermediate precision.

#### Stability

The stability of the samples was also evaluated at the level of LQC and HQC of both the analytes. The LQC were 10 and 30 ng/mL while the HQC were 120 and 1200 ng/mL for LSP and OMP, respectively. The samples were analyzed after storing at room temperature, 15 °C, and –20 °C for 24 h, and for one month at –20 °C. The sample stored at –20 °C involved freeze-thawed cycles at a gap of 12 hours between the two-consecutive freeze-thawed cycles.

#### Analytical run

The analytical run consisted of a blank sample (processed plasma sample without analytes and internal standard), zero sample (plasma sample with internal standard only), calibration standards at six levels for both the analytes (LSP and OMP plasma spiked samples with IS), low QC, medium QC, high QC, and the study samples. For acceptance of the study samples the accuracy must be within ±15% for the pharmaceutical samples [19]. In order to avoid variability all, the samples of one volunteer were analyzed together in one analytical run.

### Applicability of the method in pharmacokinetics drug-drug interaction

The developed method was applied for evaluation of pharmacokinetics drug-drug interaction between LSP and OMP in healthy human volunteers (n = 20). Both the drugs were administered to the healthy volunteers, their blood samples were collected at specified time points and analyzed for the drug content, using the developed method. Written consent form was signed by all the volunteers and purpose of the study was explained to them. They were physically examined and medical history was recorded. Age of the volunteers was 20–28 years, with mean body mass index (BMI) of 22.62 ± 2.11 lb/in^2^. Complete blood count, creatinine clearance, and liver function tests of all the healthy human volunteers were performed. All the volunteers were non-smokers and had no history of addiction. They were instructed not to take any medicines for two weeks before the study.

The study was conducted according to the “World Medical Association Declaration of Helsinki-Ethical principles of medical research involving human subjects” and its amendments. This study was approved by the ethical committee, Department of Pharmacy, University of Swabi, KP, Pakistan (Application number F.NO.UOS/2022/Pharm.02). It was an open-label, randomized, crossover, pharmacokinetic interaction study. The volunteers were divided into two groups of same size (n = 10) and the trial was conducted in two phases and two sequences, as follows;

Phase-1: First phase of the study consisted of two cycles with a wash out period of 14 days. During first phase of the study;

- **Group-I:** OMP 40 mg was administered twice a day for 6 days, and on the 7^th^ day a single dose of LSP 50 mg was administered
- **Group-II:** LSP 50 mg was administered alone as a single dose

Blood samples (3 mL) were collected at 0, 0.25, 0.5, 1, 2, 3, 4, 6, 8, 12, 24 and 36 hours. After 14 days washout period;

- **Group-I:** LSP 50 mg was administered alone
- **Group-II:** OMP 40 mg, was administered twice a day for 6 days, on the 7^th^ day LSP 50 mg was administered

Blood samples were collected at 0, 0.25, 0.5, 1, 2, 3, 4, 6, 8, 12, 24 and 36 hours

**Phase-2**:

Phase-2 of the study was started after one month of the completion of Phase-1, as follows;

**Group-I:** LSP 50 mg was given for 6 days twice a day and on the 7^th^ day single dose of OMP 40 mg was administered
**Group-II:** OMP 40 mg was administered alone.

The blood was sampled at 0, 0.25, 0.5, 1, 2, 3, 4, 6, 8, and 12 hours.

After 14 days washout period, the pattern of drug administration was reversed as follows;

**Group-I:** OMP 40 mg was administered alone.
**Group-II:** LSP 50 mg was given for 6 days twice a day and on the 7^th^ day single dose of OMP 40 mg was administered

During the study, the safety and health of the volunteers were assessed regularly by evaluation of adverse effects, vital signs, recording eating habits, and physical examination.

The pharmacokinetic parameters were determined using non-compartmental analysis using PK-Summit (version 2.0.2). The plasma concentration of the drug (ng/mL) versus time (hour) profile was constructed for both drugs when administered alone and in combination with each another. The pharmacokinetic parameters like peak plasma concentration (C_max_), time to reach peak plasma concentration (T_max_), area under the concentration-time curve from zero time to time-t (AUC_0-t_) i.e. AUC_0-36_ for LSP and AUC_0-12_ for OMP, area under the concentration-time curve from zero time to infinity (AUC_0-∞_), elimination half-life (t_1/2_) and clearance (CL) were determined.

### Statistical analysis of data

All the analysis was performed as per ICH guidelines using mean, standard deviation, and relative standard deviation. In the pharmacokinetic studies, the parameter values were compared using a *t*-test. The *P*-value (*t-test*) was calculated using the IBM SPPS statistics version 20. A p < 0.05 (95% confidence interval) was considered to be significant.

## Results

### Optimization of chromatographic conditions

Sharpsil C8 column (4.6 × 250 mm, 5 μm), Ultisil C8 column (4.6 mm × 150 mm, 5 μm) and Agilent C18 column (4.6 × 250 mm, 5 μm) were evaluated as stationary phase. The best resolution was achieved with Agilent C18 column (4.6 x 250 mm, 5 μm). Different mobile phase compositions such as methanol and water, in ratios of 60:40, and 70:30 (v/v), were used and observed the separation and characteristics of the peak. Phosphate buffer, methanol, and acetonitrile were also evaluated in the ratio of 50:40:10 and 60:30:10, with pH 6.8, and pH 7.2. The best symmetry of peaks with optimum retention times was achieved using acetonitrile and phosphate buffer, pH 7.2, in a ratio of 60:40 (v/v), respectively. The mobile phase was pumped at different flow rates (0.8 to 1.4 mL/min). Omeprazole has a strong chromophore and can give response at multiple wavelengths while in this study the focus was on levosulpiride to find a wavelength at which it gives best response. After analyzing different wavelength 280 nm was found to be the best wavelength at which both levosulpiride and omeprazole gave optimum response.

**Fig 1:**
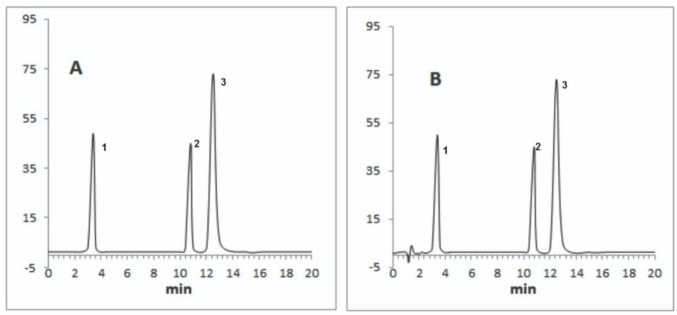
Chromatograms showing peaks of levosulpiride 50 ng/ml (1), pantoprazole the internal standard 200 ng/ml (2), and omeprazole 500 ng/ml (3) where A is a sample prepared in the mobile phase and B is sample spiked with blank human plasma.

The best peak shapes, resolution, and low HPLC pump pressure were obtained at the flow rate of 1 mL/min and it was selected for further analysis. Fig 1 shows representative chromatograms of the levosulpiride and omeprazole prepared in mobile phase (standard) and spiked human plasma sample at the optimum conditions.

### Method validation

The method was validated in terms of selectivity, specificity, linearity, sensitivity, accuracy, and precision. The stability of the extracted (spiked) samples was performed and carry-over effects was also observed.

#### Selectivity and specificity

There were no extraneous peaks in the blank spiked plasma of healthy human volunteers. The volunteer’s plasma sample collected after zero min contained two extraneous peaks having retention times of 5.4 and 8.9 min, respectively. Previously, it has been reported that these two peaks are metabolites of OMP (5-hydroxy omeprazole and omeprazole sulfone) [20]. In our previously reported study these two metabolites were quantified using HPLC-UV [21]. Both the metabolites are reported to be pharmacologically inactive [22]. All the peaks were well resolved. The chromatogram obtained after analyzing human samples obtained from healthy human volunteers was used to determine the resolution. The resolution between two consecutive peaks was as follows;

- Peak 1 and 2; 2.86
- Peak 2 and 3; 4.38
- Peak 3 and 4; 2.29
- Peak 4 and 5; 2.44

**Fig 2:**
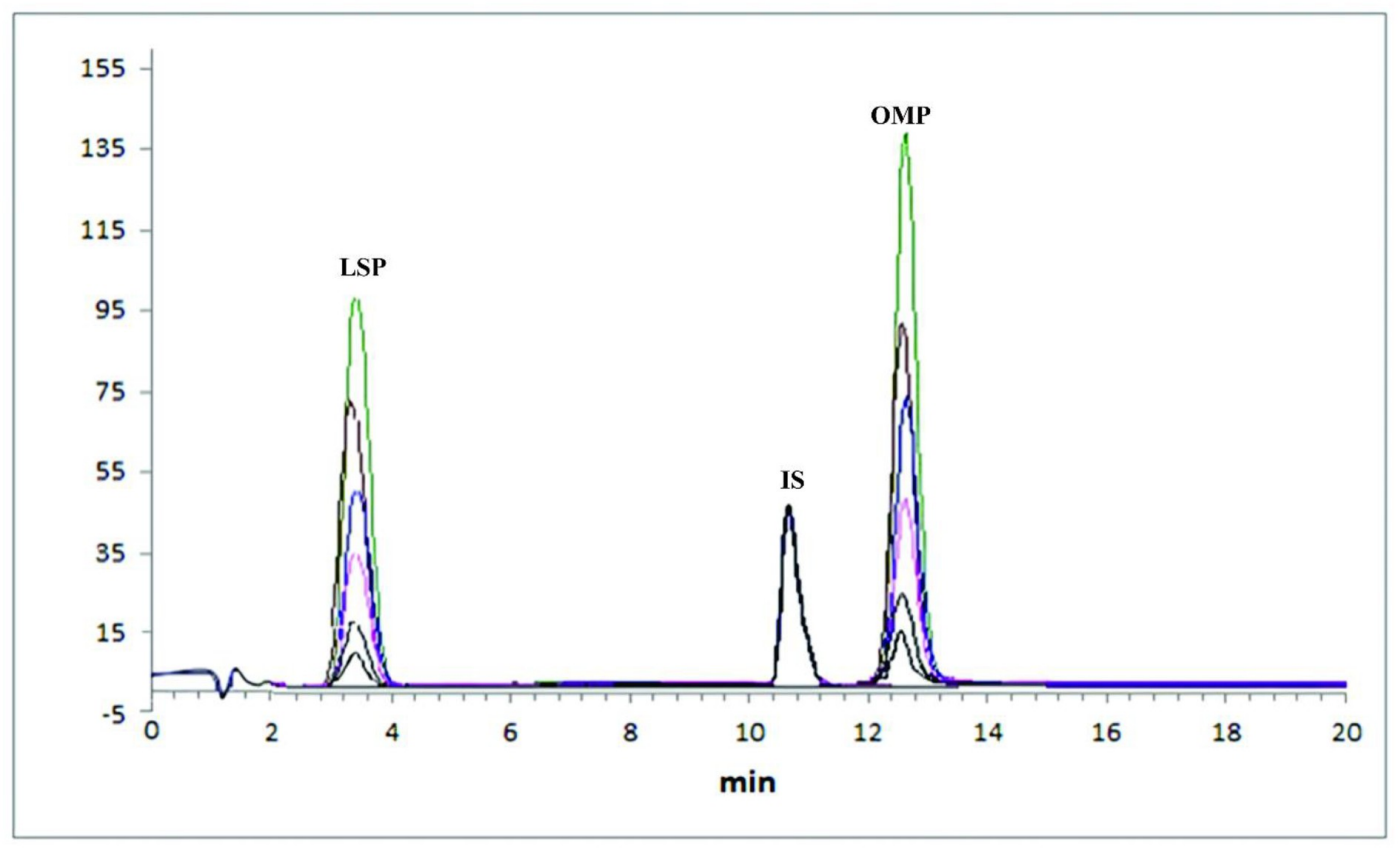
Overlay of chromatograms for both levosulpiride (LSP), internal standard (IS), and omeprazole (OMP) ranging from LLOQ to ULOQ (upper limit of quantification) in spiked plasma samples.

Apart from the two extraneous peaks no other interfering peaks were observed neither in the blank matrix (human plasma) and samples collected from human volunteers at different time intervals. The retention times and peak shapes (no peak broadening) of the analytes under study were consistent, thus confirming peak purity. The overlay of all chromatograms of spiked samples are shown in Fig 2. It also includes blank spiked plasma sample.

#### Linearity

**Fig 3:**
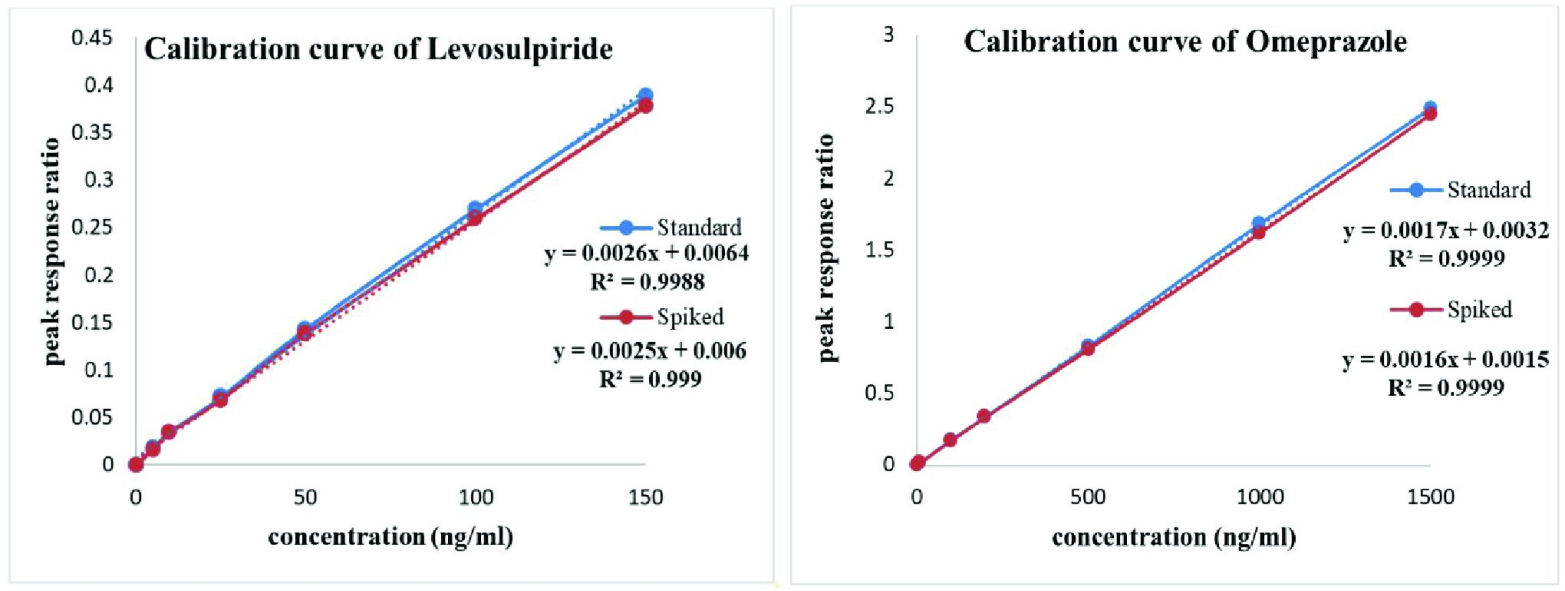
Calibration curve of standard and spiked samples of levosulpiride and omeprazole showing equation for the straight line (y = a + bx) and correlation coefficient (r^2^).

Six different concentrations of LSP (5–150 ng/mL) and OMP (10–1500 ng/mL) were used for the evaluation of linearity. The regression equation (y = a +bx) and correlation coefficient (r^2^) were calculated for both pharmaceutical and biological samples of LSP and OMP and calibration curves are shown in Fig 3.

**Fig 4:**
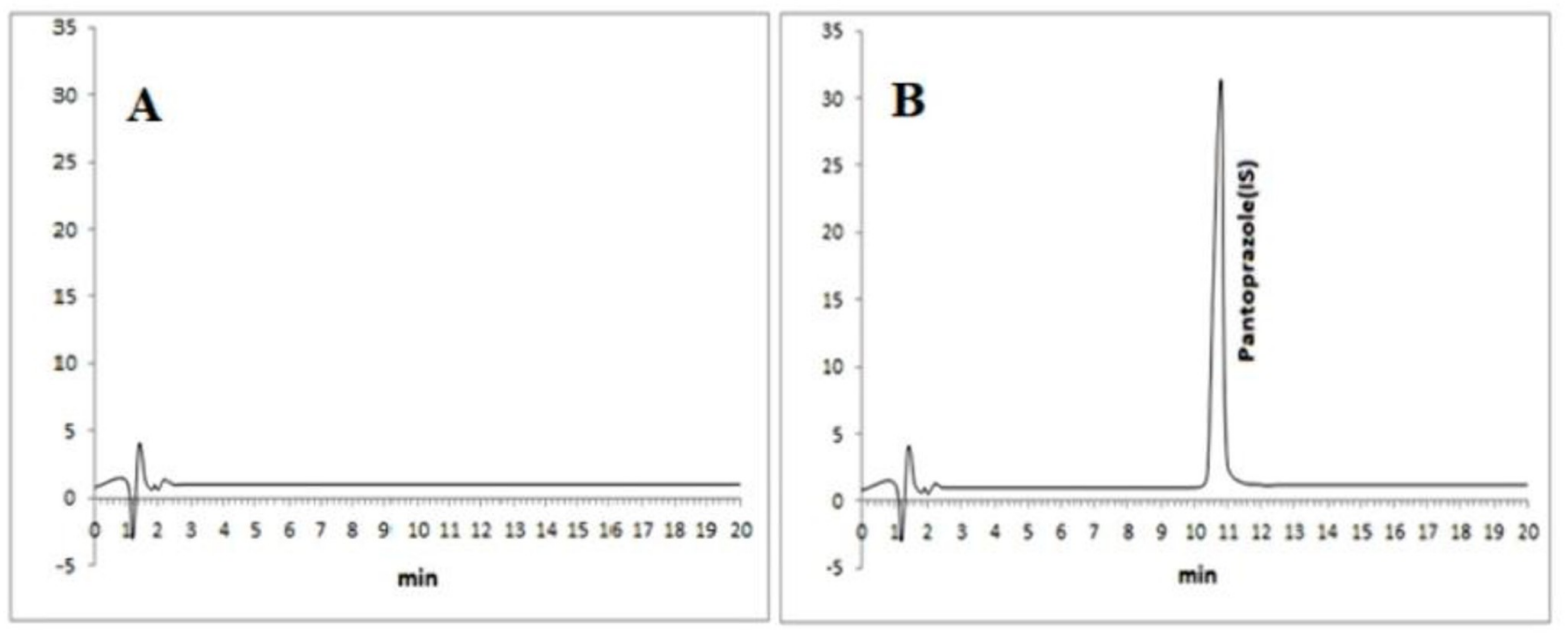
Chromatograms A) a blank plasma sample B) zero sample (a blank sample spiked only with internal standard).

**Fig 5:**
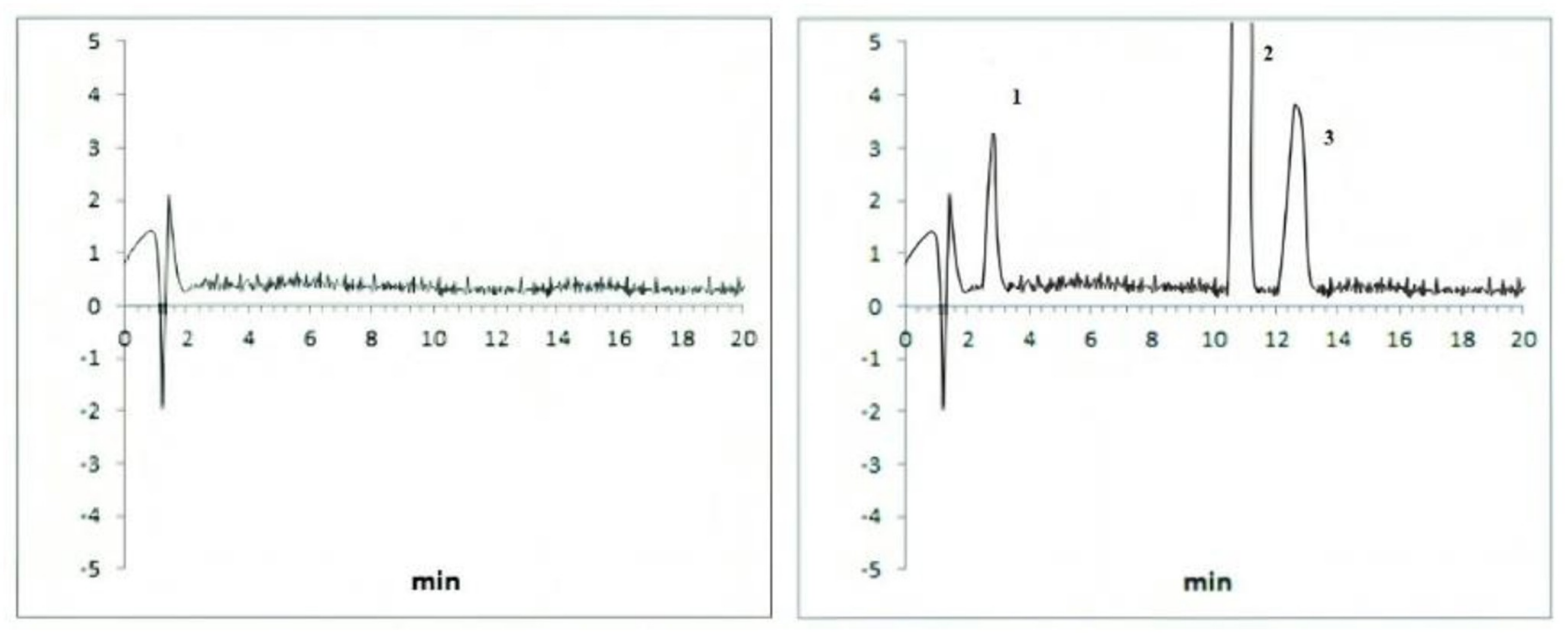
A blank spiked chromatogram and spiked chromatogram of levosulpiride (1) and omeprazole (3) at lower limit of qunatification.

The LLOQ and LOD for LSP were 5 and 2 ng/mL, respectively, while for OMP the values were 10 and 3 ng/mL. The calibration range also included blank plasma and zero samples. The chromatograms of blank and zero samples are shown in Fig 4, while the chromatograms of spiked LLOQ level for both levosulpiride and omeprazole are given in Fig 5.

The calibration standards (spiked samples) were back-calculated and are presented in Table 1. The data was collected over three separate analytical runs. Furthermore, the % intercept was calculated for the spiked samples which was 0.25 and 0.016 for LSP and OMP, respectively.

**Table 1:** Back-calculation of the calibration standards of levosulpiride and omeprazole from three analytical runs.

Levosulpiride					
Nominal Conc.(ng/ml)	Calculated mean (ng/ml)	±SD	% RSD	% error	% recovery
**5**	5.16	0.19	3.59	3.2	103.2
**10**	10.14	0.61	6.05	1.39	101.39
**25**	25.22	0.31	1.22	0.88	100.88
**50**	48.94	2.27	4.63	-2.11	97.88
**100**	98.63	2.60	2.64	-1.37	98.62
**150**	147.49	4.60	3.12	-1.67	98.33
**Omeprazole**					
**10**	10.26	0.48	4.71	2.55	102.54
**100**	98.96	3.57	3.61	-1.03	98.96
**200**	197.67	7.33	3.71	-1.17	98.83
**500**	487.71	8.50	1.74	-2.46	97.54
**1000**	988.41	16.38	1.66	-1.16	98.83
**1500**	1511.81	12.78	0.85	0.79	100.78

#### Accuracy

The accuracy was determined by the percent recovery of both the analytes. Samples having different drug concentrations were analyzed. At each concentration, the percent recovery was within the acceptable limits (±15%), as defined by ICH guidelines [19]. Details of percent recovery are presented in Table 2.

**Table 2:** Accuracy and precision of RP-HPLC method for levosulpiride and omeprazole.

Levosulpiride nominal concentration	% recovery	Omeprazole nominal concentration	% recovery
	Mean ± SD; %RSD (n = 5)		Mean ± SD; %RSD (n = 5)
5 ng/mL	102.36 ± 2.37;2.32	10 ng/mL	103.04 ± 1.91; 1.84
10 ng/mL	101.2 ± 1.17;1.15	30 ng/mL	100.33 ± 6.18; 6.16
50 ng/mL	99.74 ± 1.37;1.38	500 ng/mL	97.24 ± 2.98; 3.06
120 ng/mL	98.83 ± 2.15;2.17	1200 ng/mL	98.22 ± 1.84;1.87
**Precision**			
**Reinjection reproducibility**			
**Peak area**			
**Levosulpiride**		**Omeprazole**	
10 ng/mL	8746 ± 377;4.313	30 ng/mL	11047 ± 511;4.63
50 ng/mL	36851 ± 834;2.26	500 ng/mL	207476± 4266;2.06
120 ng/mL	88514 ± 1399;1.58	1200 ng/mL	520725 ± 5985;1.15
**Retention time (min)**			
3.53 ± 0.05; 1.63		12.57 ± 0.11; 0.92	
**Intraday repeatability**	Levosulpiride		Omeprazole
	Concentration recovered		Concentration recovered
5 ng	5.12 ± 0.119;2.32	10 ng	10.30 ± 0.21;2.15
10 ng	10.12 ± 0.121;1.15	30 ng	30.10 ± 1.85;6.16
50 ng	49.87 ± 0.69;1.38	500 ng	486 ± 14.87;3.06
120 ng	118.6 ± 2.58;2.17	1200	1178 ± 22.09;1.87
**Inter day repeatability**			
5 ng	5.07 ± 0.126;3.2.49	10 ng	10.15 ± 0.16;1.53
10 ng	10.01 ± 0.215;2.15	30 ng	29.80 ± 1.33;4.45
50 ng	48.66 ± 0.883;1.82	500 ng	476 ± 11.279;2.36
120 ng	117.80 ± 1.83;1.56	1200 ng	1171 ± 24.59; 2.11

#### Precision

The repeatability study (injection repeatability) in terms of peak areas and retention time was good and there was not much variation in the repeated analyses. The %RSD for OMP at low QC (10 ng) was slightly higher than other readings but remained within the acceptable limits. Inter day and intraday analysis for the amount (concentration) of drug recovered from the spiked sample was used for intermediate precision and the values were in complete harmony with one another.

During the validation of the method, the carry-over effect was also evaluated by analyzing a blank sample after the injection of the ULOQ sample. No carry-over effect was observed during method validation. The criteria for the analytical run were also fulfilled as the accuracy of the QC samples was within ± 15%.

#### Stability

The stability of the samples was evaluated for short term and relatively long term (one month). Two concentrations (LQC and HQC) were used for LSP and OMP as shown in Table 3. The samples showed good stability and the concentrations recovered were within limits i.e. ±15% of nominal concentration as described in ICH guidelines. The % recovery was calculated by comparing the % recovery (concentration recovered) with freshly prepared spiked samples.

**Table 3:** Stability of samples at different temperatures and at the level of LQC and HQC.

Storage condition	% recovery							
	Levosulpiride				Omeprazole			
	Low QC (10ng/ml)		High QC (120 ng/ml)		Low QC (30 ng/ml)		High QC (1200 ng/ml)	
	mean	% RSD	mean	% RSD	mean	% RSD	mean	% RSD
RT for 24 hours	96.80	3.13	98.00	1.98	96.11	3.83	98.35	1.59
15 °C for 12 hours*	98.36	2.60	99.67	1.55	98.66	3.70	99.31	1.36
-20 °C for 24 hours	98.75	2.92	99.17	2.76	99.33	2.77	99.48	1.03
-20°C for one month**	96.90	3.58	98.33	1.93	98.73	2.68	98.80	0.77

RT; room temperature;

*autosampler temperature;

**three freezed cycles involved

**Fig 6:**
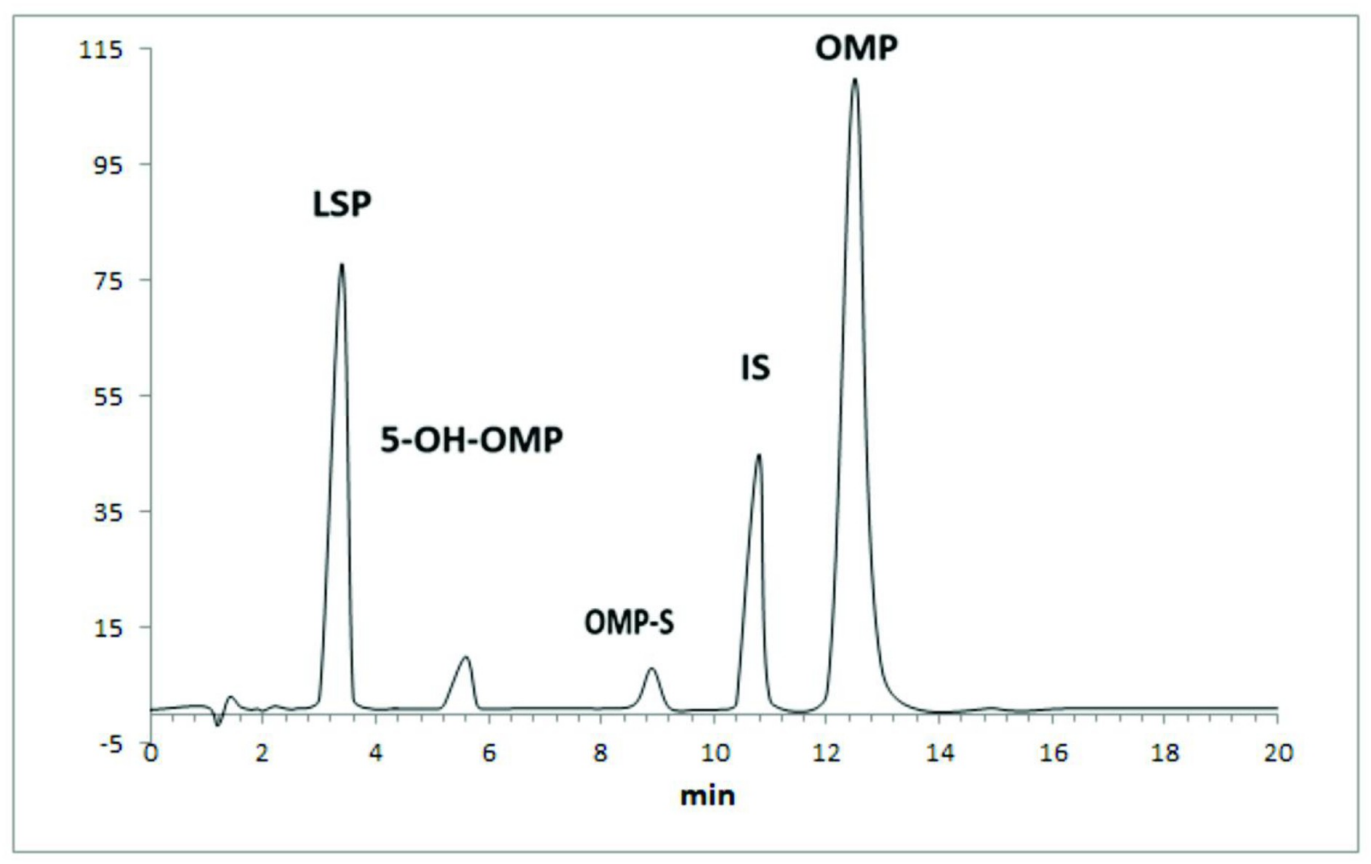
Chromatogram of a plasma sample collected from a healthy human volunteer after 4.0 hours of levosulpiride with omeprazole, where LSP is levosulpiride, 5-OH-OMP, and OMP-S are metabolites of omeprazole, IS is internal standard (pantoprazole) and OMP is omeprazole.

The method was successfully utilized for quantification of both the analytes in plasma samples collected from healthy volunteers at different time intervals. However, the volunteer’s sample contained two extraneous peaks which are the metabolites of omeprazole i.e. 5-hydroxy omeprazole and omeprazole sulfone. Fig 6 shows the chromatogram of a blood sample (plasma) obtained from a healthy volunteer after drug administration.

### Applicability of the method in evaluation of pharmacokinetics drug-drug interaction between LSP and OMP

**Fig 7:**
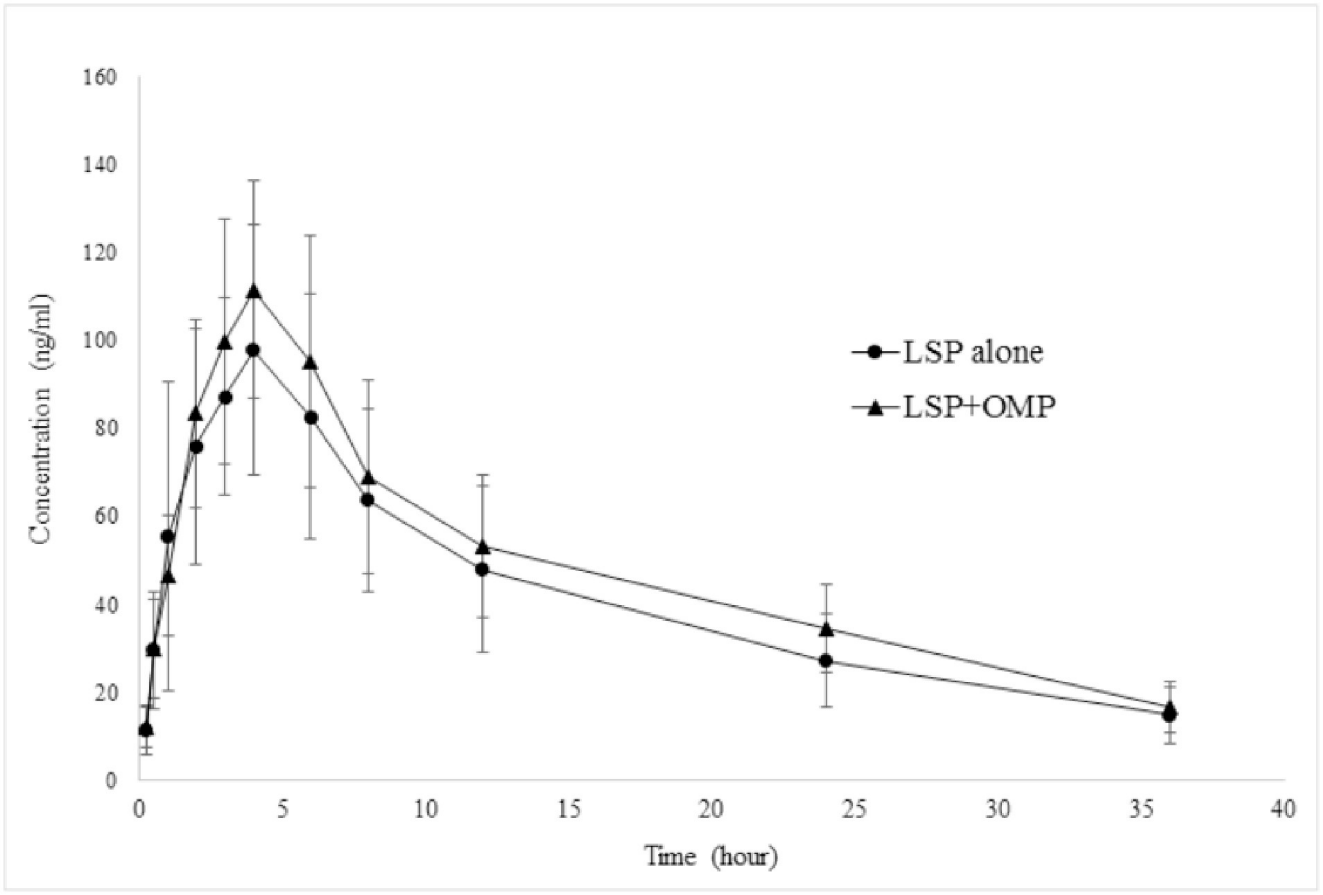
Plasma drug concentration vs time profile of levosulpiride 50 mg in healthy human volunteers (n = 20) when administered alone (LSP alone) and co-administered (LSP+OMP) with omeprazole 40 mg.

The HPLC method was used for the quantification of both the analytes in samples collected from healthy volunteers for evaluation of the pharmacokinetics drug-drug interaction between LSP and OMP. The concentration-time profile of LSP, when administered alone and co-administered with OMP is shown in Fig 7. C_max_, AUC_0-t_, and AUC_0-∞_ were increased while CL was reduced (Table 4).

**Table 4:** Pharmacokinetic parameters of levosulpiride 50 mg in healthy human volunteers (n = 20) when administered alone and in combination with omeprazole 40 mg.

Pharmacokinetic parameter	Levosulpiride when administered alone (mean ± SD)	Levosulpiride administered with omeprazole (mean ± SD)	P-value
C_max_ (ng/mL)	108.61 ± 24.95	118.151 ± 26.31	0.260
AUC_0-t_ (ng-hr/mL)	1542.36 ± 473.46	1766.000 ± 481.51	0.156
AUC_0-∞_ (ng-hr/mL)	1870.21 ± 626.792	2213.075 ± 576.51	0.087
T_max_ (hr)	3.80 ± 1.055	3.712 ± 0.75	0.707
t_1/2_ (hr)	13.29 ± 2.642	12.714 ± 4.76	0.718
CL/F(mL/hr)	35832 ± 8402	30333 ± 6326	0.056

The C_max_, AUC_0-t_, and AUC_0-∞_ were increased by 8.79%, 14.52%, and 18.36% respectively while CL was reduced by 15.34%. None of the pharmacokinetic parameters of LSP were altered by OMP significantly.

**Fig 8:**
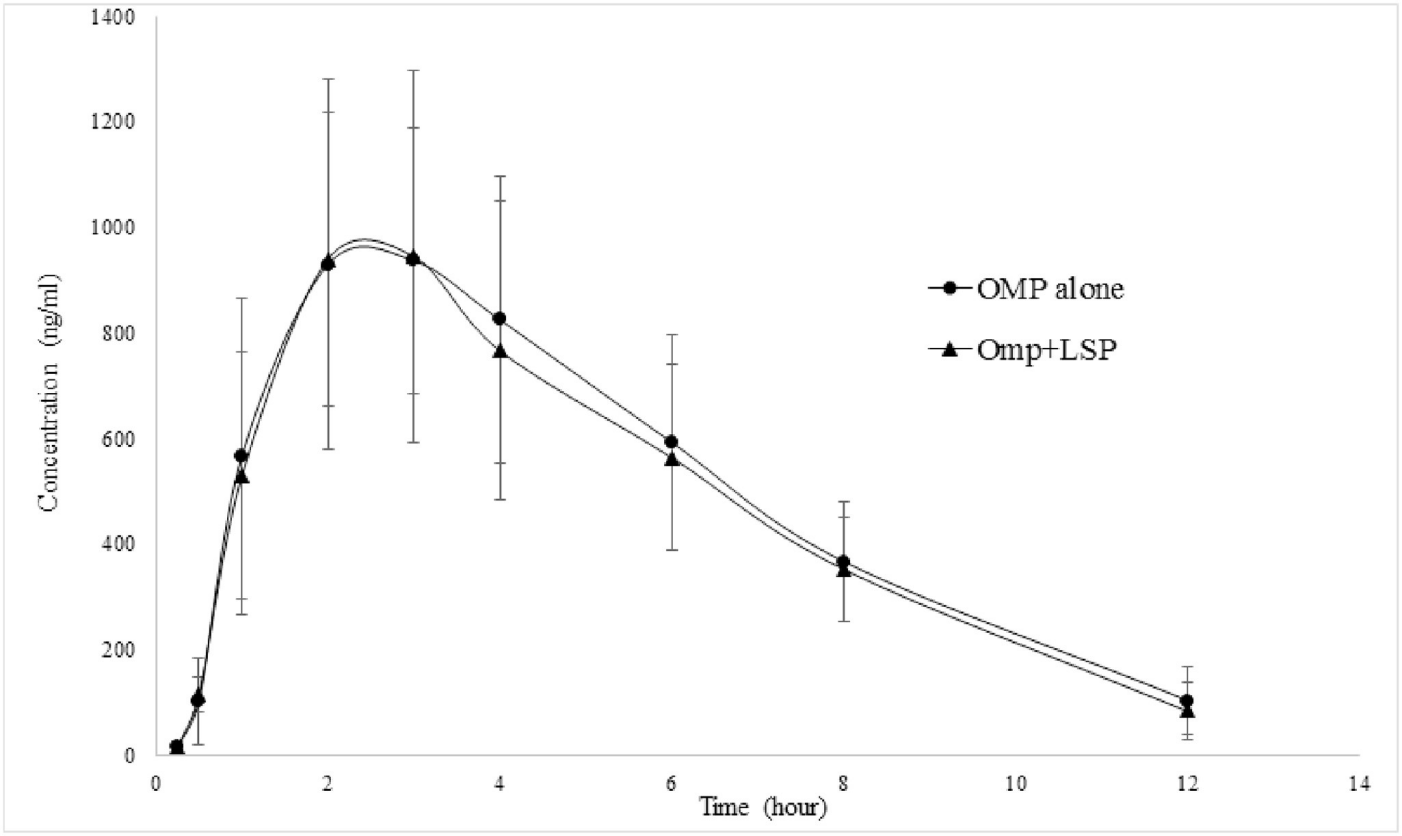
Plasma drug concentration vs time profile of omeprazole 40 mg in healthy human volunteers (n = 20) when administered alone (OMP alone) and co-administered (OMP+LSP) with levosulpiride 50 mg.

After a gap of a month, the second phase of the study was conducted to evaluate the effect of LSP on OMP. The blood samples were collected at different time of intervals up to 12 hours (the sampling time for OMP was reduced due to its shorter half-life compared to LSP). The drug concentration versus time profile of OMP alone, and in combination with LSP is presented in Fig 8.

The pharmacokinetics of OMP were not altered significantly by co-administration with LSP (Table 5). The parameters C_max_, AUC_0-t_, AUC_0-∞_, t_1/2_, and CL were decreased by 1.76%, 2.31%, 2.45%, and 0.25%, respectively. None of the pharmacokinetic parameters was altered significantly.

**Table 5:** Pharmacokinetic parameters of omeprazole 40 mg in healthy human volunteers (n = 20) when administered alone and in combination with levosulpiride 50 mg.

Pharmacokinetic parameter	Omeprazole when administered alone (mean ± SD)	Omeprazole administered with Levosulpiride (mean ± SD)	*p*-value
C_max_ (ng/mL)	1142.35 ± 261.57	1122.17 ± 258.38	0.812
AUC_0-t_ (ng-hr/mL)	6092.75 ± 1477.57	5951.61 ± 1427.45	0.766
AUC_0-∞_(ng-hr/mL)	6652.91 ± 1646.41	6489.67 ± 1608.69	0.762
T_max_ (hr)	1.95± 0.99	1.85 ± 0.83	1.0
t_1/2_ (hr)	2.30 ± 0.94	2.23 ± 1.034	0.752
CL/F (mL/hr)	7118.71 ± 2407.41	7100.69 ± 2001.55	0.980

## Discussion

LSP and OMP both have different physico-chemical characteristics and development of an isocratic HPLC-UV method for their simultaneous analysis was a challenging task. LSP has been conveniently quantified in human plasma by LCMS [17] and HPLC with a fluorescence detector [18]. HPLC/UV methods for quantification of LSP in human plasma samples are not available. UPLC-UV method for simultaneous quantification of rabeprazole and LSP has been reported [23] but the method lacked sensitivity. In a review by Bosch et al., OMP has been quantified in different matrices by HPLC-UV at different wavelengths in the range of 280 nm to 302 nm, alone and with other analytes [24]. The evaluation and selection of mobile phase was based on previously reported studies for OMP [24] and LSP [25]. In the current study, both the drugs were analyzed at different wavelengths in the range of 220 to 320 nm. The optimum peak response of LSP, OMP and PTP (IS) was observed at 280 nm. Usually, for efficient recovery (extraction) either liquid-liquid or solid-phase extraction methods are used. In this study, the samples were extracted with a liquid-liquid extraction using ethyl acetate and dichloromethane (4:1 v/v). In comparison with solid phase extraction, liquid-liquid extraction is the most simple, efficient and rapid method. This bioanalytical method was validated according to ICH guidelines and the back-calculation of concentration from the same calibration curve was also performed as suggested in the guidelines. The method was accurate, precise and sensitive enough to conduct the pharmacokinetic studies of LSP and OMP.

The oral bioavailability of LSP is less than OMP and has a low plasma concentration. During method development LSP was more critically considered to enable quantification of the lowest possible concentration in human blood samples.

LSP is a BCS class-IV drug (having poor solubility and permeability) with low bioavailability [26]. It is a substrate of p-glycoprotein [27] and the slight increase (though non-significant) in the bioavailability of LSP and reduced clearance is the result of inhibition of p-glycoprotein by OMP [28]. The results of this study are in accordance with a previous study in which the bioavailability of itraconazole was enhanced by OMP due to p-gp inhibition [29]. However, in the current study the observed changes in the pharmacokinetics of LSP were non-significant. In the current study the elimination half-life is 13.29 ± 2.642 hours when administered alone and 12.714 ± 4.76 hours after co-administration with omeprazole and no significant change has been observed in it. The reported elimination half-life for orally administered LSP is 12.0, 12.6, and 14.3 hours for 25 mg, 50 mg, and 100 mg LSP, respectively [30]. A review by Corazza and Tonini has reported the elimination half-life to be in the range of 6 to 19 hours depending upon route of administration, dosage form and dose [31].

Higher doses and long-term use of LSP with OMP may increase the risk of parkinsonism and tardive dyskinesia [32]. Lower doses of LSP (50 mg or less) are safe and can be used in gastric functional dyspepsia, gastroparesis, and depression [33]. It is suggested to conduct long-term studies to observe pharmacodynamic effects in clinical settings when LSP and OMP are co-administered in higher doses.

OMP is a substrate and inhibitor of p-gp, CY2C19, and CYP3A4. It has been used as a probe drug for CYP2C19 activity. While LSP is a substrate of p-gp, and can’t induce or inhibit its activity. Its CYP450 enzyme system activity has not been reported. In the present study, the second phase of the interaction study, LSP did not alter the pharmacokinetic parameters of OMP. The non-significant pharmacokinetic interaction of LSP with OMP authenticated that LSP had no role in induction or inhibition of p-gp, CYP2C19, and CYP3A4. The Cmax and AUC of OMP were not significantly decreased by LSP but a study reported a decrease in the Cmax of rabeprazole by a prokinetic agent [34]. However, there is another study that demonstrated an increase in C_max_ and AUC of a PPI (rabeprazole) with co-administration of a prokinetic agent (mosapride) [6]. The differences in the pharmacokinetics of OMP in the current study and the previous studies may be due to the different populations, a different drug of the PPI class, and/or variability in genetic polymorphism. A pharmacokinetic drug-drug interaction study between esomeprazole and apixaban has been conducted in rats. Esomeprazole a p-gp inhibitor significantly increased the bioavailability of apixaban while esomeprazole bioavailability was significantly decreased by apixaban co-administration. The reported study in rats did not provide any authentic reason (a mechanism) for a decrease in esomeprazole bioavailability [35].

## Conclusion

LSP and OMP are co-prescribed for gastrointestinal disturbances and related psychological problems without any dose adjustments. A reversed-phase HPLC/UV method was developed and validated for the simultaneous quantification of LSP and OMP. The method was optimized and validated as per standard guidelines. The method was sensitive and linear for both the analytes. Plasma samples demonstrated good stability when stored at -20 °C for one month. The developed method was applied for evaluation of pharmacokinetics drug-drug interaction of LSP and OMP. This study confirmed that p-gp inhibition by OMP increased the AUC and decreased the CL/F of LSP. But the observed changes in pharmacokinetics of LSP were insignificant and it was safe to co-administer LSP (50 mg) and OMP (40 mg). Pharmacokinetics of OMP were not affected by LSP and dose adjustment was not needed. However, further studies are required if higher doses of LSP are used for longer time.
